# Golgi apparatus dis- and reorganizations studied with the aid of 2-deoxy-d-glucose and visualized by 3D-electron tomography

**DOI:** 10.1007/s00418-016-1515-7

**Published:** 2016-12-14

**Authors:** Carmen Ranftler, Claudia Meisslitzer-Ruppitsch, Josef Neumüller, Adolf Ellinger, Margit Pavelka

**Affiliations:** Center for Anatomy and Cell Biology, Medical University of Vienna, Schwarzspanierstraße 17, 1090 Vienna, Austria

**Keywords:** Golgi apparatus, 2-Deoxy-d-glucose (2DG), Golgi dynamics, 3D-electron tomography

## Abstract

We studied Golgi apparatus disorganizations and reorganizations in human HepG2 hepatoblastoma cells by using the nonmetabolizable glucose analogue 2-deoxy-d-glucose (2DG) and analyzing the changes in Golgi stack architectures by 3D-electron tomography. Golgi stacks remodel in response to 2DG-treatment and are replaced by tubulo-glomerular Golgi bodies, from which mini-Golgi stacks emerge again after removal of 2DG. The Golgi stack changes correlate with the measured ATP-values. Our findings indicate that the classic Golgi stack architecture is impeded, while cells are under the influence of 2DG at constantly low ATP-levels, but the Golgi apparatus is maintained in forms of the Golgi bodies and Golgi stacks can be rebuilt as soon as 2DG is removed. The 3D-electron microscopic results highlight connecting regions that interlink membrane compartments in all phases of Golgi stack reorganizations and show that the compact Golgi bodies mainly consist of continuous intertwined tubules. Connections and continuities point to possible new transport pathways that could substitute for other modes of traffic. The changing architectures visualized in this work reflect Golgi stack dynamics that may be essential for basic cell physiologic and pathologic processes and help to learn, how cells respond to conditions of stress.

## Introduction

The Golgi apparatus in mammalian cells is a complex and dynamic organelle that is involved in multiple cellular tasks and built up from interconnected highly organized stacks of cisternae that form a central crossroads in both the secretory and the endocytic pathways (Farquhar and Palade 1981; Mollenhauer and Morré 1991; Berger 1997; Roth 1997; Farquhar and Palade 1998; Lippincott-Schwartz et al. 1998; Pavelka et al. 2008; Klumperman 2011; Lowe 2011; Nakamura et al. 2012; Day et al. 2013; Pavelka and Roth 2013; Papanikou and Glick 2014; Rothman 2014). Its structures and functions have been extensively studied, and advanced microscopic methods, including cryo-techniques and correlative light-electron microscopy, together with three-dimensional reconstructions have provided better insights into the complex Golgi architectures and fine structure–function relationships (e.g., Ladinski et al. 1999; Grabenbauer et al. 2005; Han et al. 2013; Marsh and Pavelka 2013; Beznoussenko et al. 2014; Engel et al. 2015; Koga et al. 2016). On the other hand, many questions are unanswered, and the large number of controversial results obtained with respect to the organization of the Golgi apparatus is confusing. To a large part, the difficulties in the exploration of the Golgi organization are due to the high dynamics of the organelle and the rapid reorganizations occurring concomitantly with functional changes, under pathological conditions, during the cell cycle and in response to environmental changes and drug treatments (e.g., Rabouille and Warren 1997; Dinter and Berger 1998; Vetterlein et al. 2003; Mironov et al., 2004; Wang and Seemann 2011; Villeneuve et al. 2013; Dong et al. 2014; Haase and Rabouille 2015; Machamer 2015; Kaneko et al. 2016; Rabouille and Haase 2016). The present work aims to improve the insight into Golgi apparatus dynamics and unravel processes of Golgi apparatus disorganizations and reorganizations. For this, we used 2-deoxy-d-glucose (2DG), a nonmetabolizable glucose analogue, which causes a reversible decrease of the cellular levels of adenosine triphosphate (ATP) and which is used in both experimental cell biology and medicine (e.g., Aft et al. 2002; Dwarakanath 2009; Kavaliauskiene et al. 2015; Zhang et al. 2015). Since the Golgi apparatus changes its architecture under the influence of 2DG, this drug was found to be of great value in the context of Golgi apparatus research (del Valle et al. 1999; Meisslitzer-Ruppitsch et al. 2011; Ranftler et al. 2015).

The sugar 2-deoxy-d-glucose (2-deoxy-d-arabino-hexose) is the 2-deoxy derivate of d-glucose and d-mannose. Its synthesis from d-glucose has already been described more than 90 years ago by Bergmann and colleagues (Bergmann et al. 1922, 1923). During the past decades, 2DG has been much used in basic research and for medical applications. It has a wide range of effects, which include anti-viral and anti-tumor response, both tested in vitro and in vivo (e.g., Aft et al. 2002; Kang and Hwang 2006; Dwarakanath 2009; Diaz-Ruiz et al. 2011; Raez et al. 2013; Zhang et al. 2014). 2DG is a potential drug for the treatment of epilepsy owing to its anti-convulsant properties (e.g., Stafstrom et al. 2009; Ockuly et al. 2012) being well tolerated at effective doses. Its capability of inducing ketogenesis and supporting or mimicking a ketogenic diet is important for the treatment of epilepsy (Garriga-Canut et al. 2006), as well as other diseases, such as Morbus Alzheimer (Yao et al. 2011) or malignant astrocytoma (Marsh et al. 2008). Moreover, 2DG has been found to enhance autophagy, or have discriminating effects on autophagy regulation (Wu et al. 2009; Wang et al. 2011; Xi et al. 2011; Jeon et al. 2015), another fact that is of particular interest in anti-tumor-application. The combined use of 2DG and cytostatic drugs and other substances offers promising new possibilities in tumor therapies (Estañ et al. 2012; Huang et al. 2013; Mustafa et al. 2015; Oladghaffari et al. 2015; Li et al. 2015; Jangamreddy et al. 2015; Zhang et al. 2015). 2DG might also help in the treatment of systemic lupus erythematodes by normalizing the T cell metabolism (Yin et al. 2015) and has been recognized as a caloric restriction mimetic (Ingram and Roth 2015). It has recently been shown that 2DG alters the levels and species compositions of several lipids; it becomes incorporated into the carbohydrate moiety of glycosphingolipids and protects cells against Shiga toxins (Kavaliauskiene et al. 2015).

Based on its chemical structure, 2DG acts by influencing N-glycosylation (d-mannose analogue) and glycolysis (d-glucose analogue), thereby changing the cells’ energy level by decreasing the amount of intracellular ATP (del Valle et al. 1999; Ranftler et al. 2015) and altering the structures of cell organelles and the endomembrane system (del Valle et al. 1999; Grieb et al. 2004; Wu et al. 2009; Meisslitzer-Ruppitsch et al. 2011; Ranftler et al. 2015). Although 2DG has become a drug of high scientific and medical interest, only few reports have addressed its action with regard to the ultrastructure of cells. Previous studies in our laboratory (Meisslitzer-Ruppitsch et al. 2011; Ranftler et al. 2015) have shown that treating cultured cells with 2DG leads to extensive changes of the Golgi apparatus. In the present work, we followed the dissociations and re-formations of the Golgi stacks that occur under the influence of 2DG and after administration of the drug had ended. We asked, whether these reorganizations are correlated with the changes in the cellular ATP-levels. Both chemically fixed and high-pressure frozen cells were analyzed ultrastructurally, and the Golgi apparatus architectures were studied three-dimensionally by using electron tomography. Initial Golgi apparatus reorganizations start few minutes after onset of 2DG treatment simultaneously with a rapid depression of the cells’ ATP-levels. Continued treatment with 2DG leads to the disappearance of regular Golgi apparatus stacks, which are replaced by vesiculo-tubulo-glomerular Golgi bodies. All these changes are completely reversible, which makes the substance best suitable for a close observation of the dynamics of the Golgi apparatus during disorganizations and reorganizations of the stacks; the results also help to understand the way, in which cells respond to the conditions of stress, which is particularly relevant, when 2DG is used in experimental cell biology and medical sciences, and is applied clinically.

## Materials and methods

### General cell culture procedures

HepG2 cells (epithelial hepatoblastoma cells, HB-8065, ATCC) were grown in Minimum Essential Medium with Eagle salts (MEM) containing 10% Fetal Bovine Serum (FBS), 2 mM l-glutamine and 1% NonEssential Amino Acid Solution (NEAA) in a 95% humidified atmosphere with 5% CO_2_ and a constant temperature at 37 °C. All agents were purchased from PAA Laboratories GmbH, Pasching, Austria.

### Experimental settings

To provide an experimental basis for correlative studies of morphologies and fine structures of cells and their ATP-concentrations, we designed an assembly, which enabled us to use the same cultures for combined microscopic analyses of chemically fixed and high-pressure frozen cells as well as for ATP-measurements (Ranftler et al. 2013). Petri dishes, each containing a glass cover slip with a duplet of sapphire disks positioned on its surface, were prepared before the seeding of the cells. The cells grown on the bottom of the Petri dishes were used to measure the intracellular ATP-levels, while the cells on the glass cover slips were chemically fixed and the cells on the sapphire disks were taken for high-pressure freezing (HPF). Both chemically and HPF fixed cells were further prepared for electron microscopy and electron tomography.

### Treatments with 2DG

HepG2 cell cultures (growing either on glass coverslips, sapphire disks or Petri dishes) were used for experiments 48 h after seeding and 24 h after a medium change to Dulbecco’s Modified Eagles Medium containing 1 g/l glucose (DMEM; Sigma-Aldrich, St. Louis, USA), 10% FBS, 1% NEAA, 4 mM l-glutamine and 1% Antibiotic Antimycotic Solution (PAA). In this medium, cells were grown at increased CO_2_-concentrations (7.5%). At the beginning of the experiments, the cells had reached a confluence of 60–80%.

Before 2DG was applied, the HepG2 cell cultures were washed with glucose-pyruvate-free DMEM (GPF, Sigma-Aldrich) supplemented with 1% NEAA, 1% dialyzed FBS (PAA) and 4 mM l-glutamine. For ATP-depletion, the cells were incubated in GPF containing 10, 25 or 50 mM 2DG (Sigma-Aldrich) for various periods (1 min to 24 h). For the purpose of ATP-replenishment, the cells were washed with GPF and subsequently incubated for various periods (up to 5 h) in GPF alone or with 50 mM d-glucose (Sigma-Aldrich) and 1% pyruvate (Sigma-Aldrich) added.

### Measurement of the intracellular ATP-level

Adherent HepG2 cells grown on Petri dishes were used for measurements of their ATP-concentrations either untreated or following 2DG-treatment for various times, or various post-incubation periods after 2DG-removal. For this, we used the ATPlite Luminescence ATP Detection Assay System (Perkin Elmer) based on the luciferase–luciferin method, according to the guidelines of the producer. The photon emission was measured by using a luminometer (PhL Luminometer, Mediators Diagnostika, Vienna; Austria). Control values were set as 100%; treated samples were referred to these as percentages. Furthermore, a calibration curve was used to obtain the total amount of ATP in [pM].

In a single experiment, one approach was performed twice or thrice, and measurements of each sample were taken in quadruples, of which a mean value was determined. Experiments were repeated at least four times.

### Electron microscopy

#### Chemical fixation

Cells grown on glass coverslips were fixed by using 2.5% glutaraldehyde (Sigma-Aldrich) in ice-cold 0.1 M cacodylate buffer pH 7.4 (Merck) for 90 min at 4 °C. Subsequently, the cells were washed three times with 0.1 M cacodylate buffer (pH 7.4) before they were stained in osmium/potassium-ferrocyanide solution (2% OsO_4:_ 3% K_4_Fe(CN)_6_ = 1:1 vol/vol; both Merck) for 15 min at 4 °C and then in 1% veronal acetate (Merck)-buffered OsO_4_ solution for 4 h at 4 °C. Afterward, the OsO_4_ solution was discarded, and 70% ethanol (Merck) was added for 90 min at room temperature. The cells were dehydrated in a graded series of ethanol (70, 80, 96%, EtOH_absolute_) and embedded in epoxide resin (Serva, Heidelberg, Germany). After completed polymerization, 80-nm ultrathin sections for normal transmission electron microscopy or 200–300-nm-thick sections for electron tomography were prepared (UltraCut-UCT microtome, Leica Inc., Vienna, Austria) and transferred onto copper-grids (Agar Scientific Elektron Technology UK Ltd, Stansted, Great Britain). The sections were stained in 1% uranyl acetate (Merck) for 5 min. Alternatively, the sections were stained for 20 min with 0.2% OTE (Oolong Tea Extract, Nisshin EM Co. Ltd. Tokyo, Japan) in phosphate-buffered saline (PBS, Sigma-Aldrich, pH 7.4). After that, the sections were incubated for other 5 min with 8% alkaline lead citrate (Merck). Each step was performed at room temperature. Finally, the sections were examined electron microscopically at 80 kV (Tecnai-20, FEI Company, Eindhoven, The Netherlands). Digital images were recorded by using an Eagle 4k CCD camera; chip size: 4.096 × 4.096 pixels (FEI Company). Appropriate regions of interest were chosen for electron tomography.

#### High-pressure freezing

In parallel, the HepG2 cells grown on sapphire disks (BAL-TEC, Liechtenstein) were used for high-pressure freezing (BAL-TEC HPM 010 high-pressure freezing machine). Sandwiches of two sapphires facing each other with the surfaces containing the cell layers were positioned into the specimen holder. They were separated in the middle by a copper spacer and bordered by an aluminum spacer on each cell-free side of the sapphires (for illustration and step-by-step description of the process see Ranftler et al. 2013). After freezing, the specimens were stored in liquid nitrogen before substitution in a cocktail containing 1% OsO_4_ and 0.4% uranyl acetate in acetone (Merck). For preparation of the cocktail, 0.04 g uranyl acetate dihydrate was dissolved in 10 ml acetone by ultrasonication, followed by addition of 0.1 g OsO_4_. Substitution was performed in a Leica ASF system (Leica Microsystems, Vienna, Austria) for 8 h at −90 °C before raising the temperature to −60 °C within 30 min. After 8 h at −60 °C, the temperature was increased to −30 °C within 30 min and then kept constant for a further 8 h. All these steps were done by automatic devise control. Finally, the temperature was set to 0 °C within 10 min manually prior to warming up the samples to ambient temperature. Then, after rinsing the samples in acetone, they were embedded in epoxide resin. After polymerization, ultrathin sections (80 nm) for transmission electron microscopy and 200–300-nm-thick sections for electron tomography were prepared and stained as described above.

### Electron tomography

Electron tomography was performed in a Tecnai-20 electron microscope at 200 kV using either a single high-tilt holder (FEI company) or a high-tilt rotation holder (Gatan, Inc., Pleasanton, CA), which allowed adjusting the structures in optimal position. Series of tilted images within an angular range of −65° to +65° and a tilt increment of 1° were recorded automatically by the Xplore 3D software (FEI company) using a 1k Gatan slow-scan CCD camera (chip size: 1.024 × 1.024 pixels) or an Eagle 4k CCD camera (FEI Company; chip size: 4.096 × 4.096 pixels). In order to reconstruct the volume of the 200–300-nm-thick sections into virtual slices, we used the Inspect 3D software (FEI company) or the IMOD software (Boulder Laboratory for 3D Electron Microscopy of Cells, University of Colorado, USA; Kremer et al. 1996). For 3D-modeling, the structures of interest in each slice were traced with colored contours that were merged in the Z-axis with the help of the Amira 5.3 software (Mercury Computer Systems, Merignac, Cedex, France). Concerning the analysis of vesicles, only those globular membrane structures, which were fully embedded within the volume studied by electron tomography, were denoted as “vesicles.” For a more detailed analysis, some of the 3D-models were cut horizontally or vertically at different levels numbered from 1 (uppermost position in the model) to 100 (lowermost position); in Fig. 8, the positions of the cut face levels are indicated in the respective panels.

## Results

**Fig. 1:**
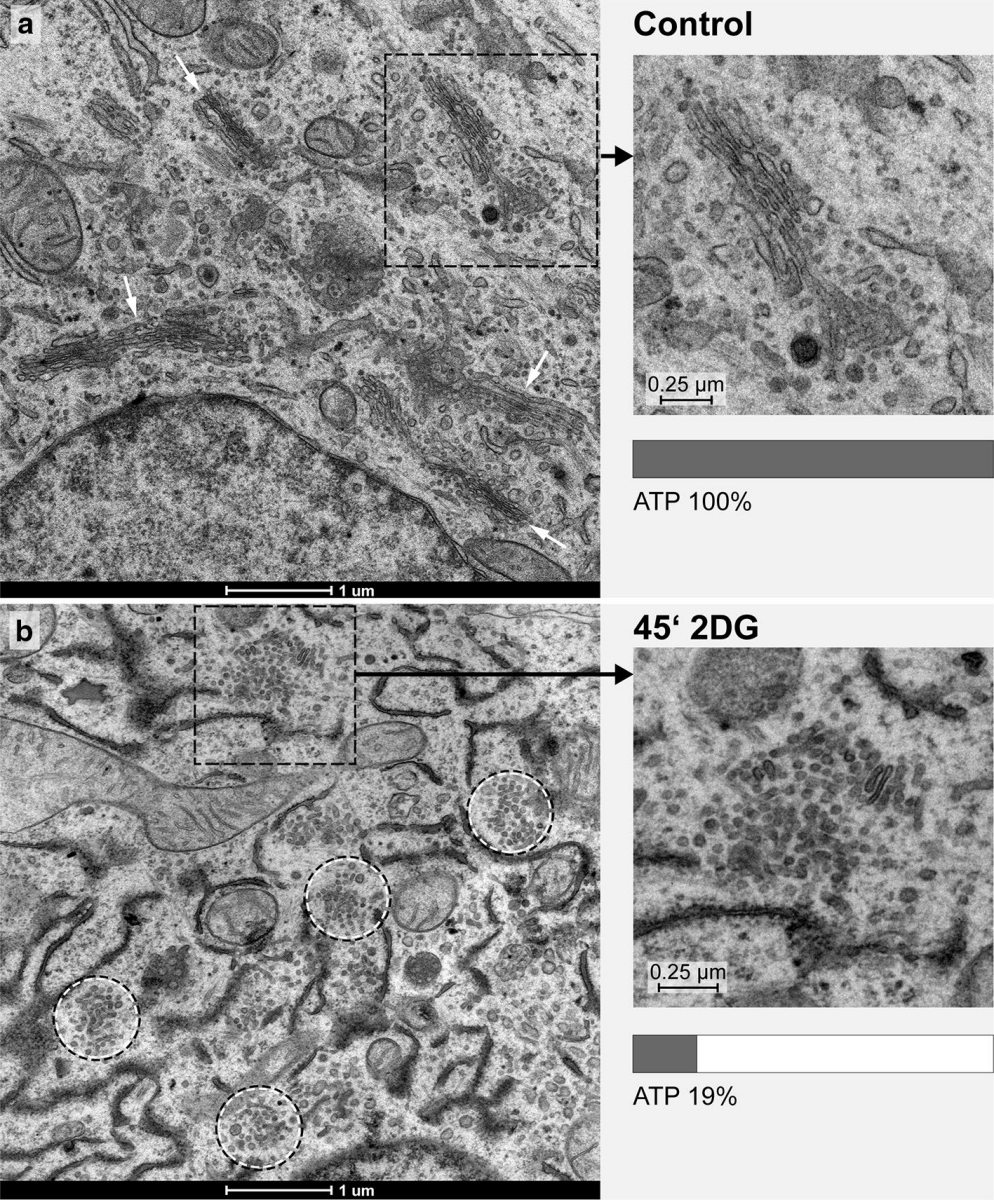
Ultrastructures of the Golgi apparatus in HepG2 hepatoma cells, high-pressure frozen, freeze substituted and embedded in Epon, are shown on thin sections of a control cell (**a**, *inset*) and after 45 min of treatment with 2DG (**b**, *inset*). In the control cell (**a**), several regular stacks of cisternae in parallel organization are apparent (*white arrows*); in the 2DG-treated cell (**b**), regular Golgi stacks are missing and instead Golgi bodies composed of irregular and loosely organized membranous compartments dominate (*circles*). In both panels, areas marked by a *rectangle* are shown in the *inset* at higher magnification and respective ATP-values are indicated. In all pictures, RER-cisternae are found close to the Golgi stacks and Golgi bodies; the RER luminal contents appear denser in the 2DG-treated cells (**b**) than in the controls (**a**)

**Fig. 2:**
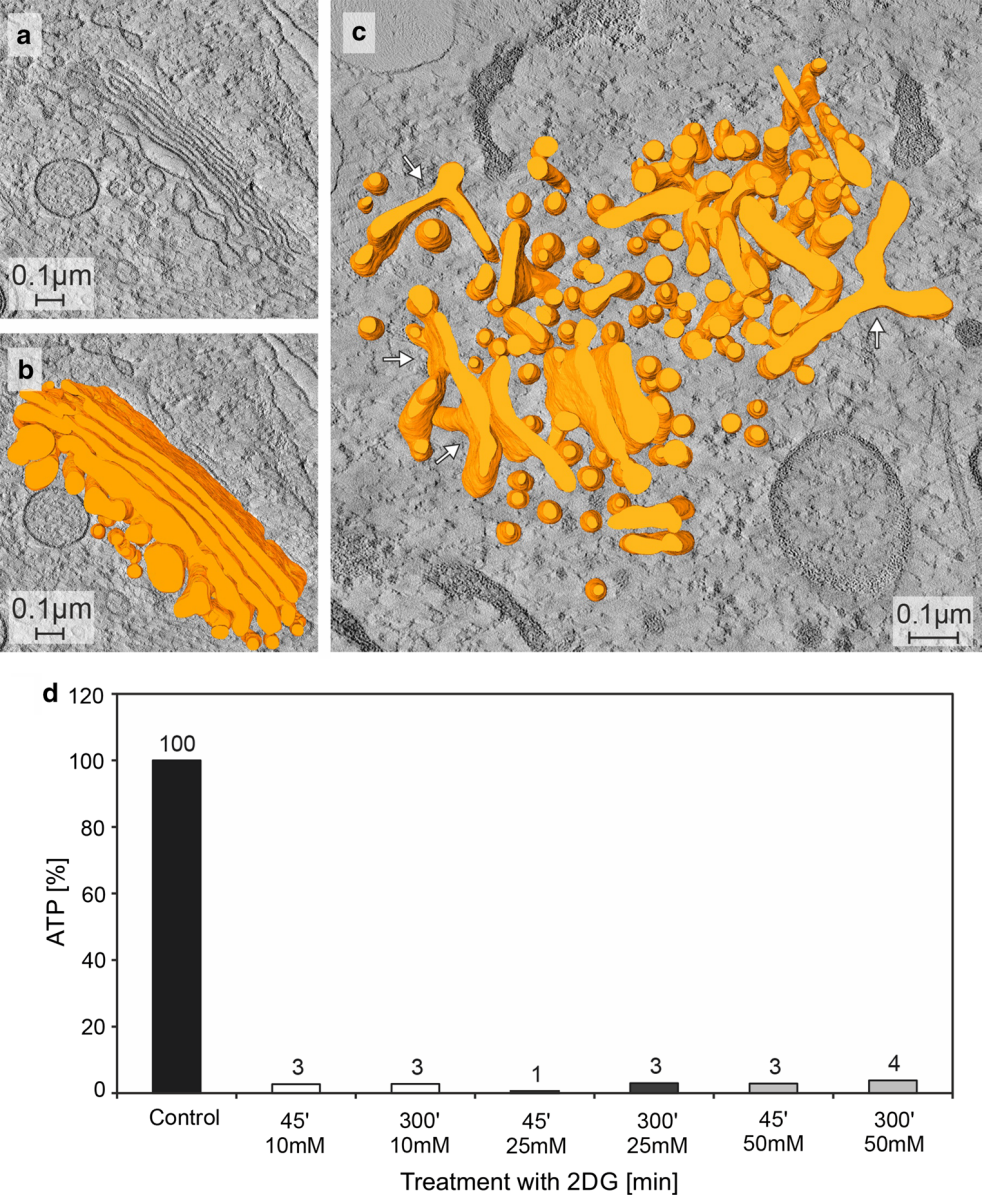
Tomographic slices and three-dimensional models of a control cell Golgi apparatus stack in **a** and **b**, and a Golgi body of a cell treated with 2DG for 45 min in **c** are shown. In both cases, the cell cultures were high-pressure frozen, freeze substituted and embedded in Epon. In contrast to the parallel organization of the cisternae that build up a Golgi stack in the control cell (**b**), the 2DG-treated cell shows various, in part tubular, cisternal and small vesicular compartments, that form a loosely arranged Golgi body (**c**). Branched and bifurcated structures (*white arrows*) are common. Volumes of the calculated tomograms (x, y, z): **a**, **b** 3.535 × 2.919 × 205 pixels, pixel size 0,59 nm; **c** 4.073 × 3.943 × 140 pixels, pixel size 0,46 nm. **d** The percentage ATP-values after 45 min and 5 h of treatment of HepG2 cell cultures with 2DG of different, 10, 25 and 50 mM, concentrations. The graphic shows that a massive reduction of the ATP-levels is obtained with each of the 2DG-concentrations tested. The data shown are taken from one representative experiment; comparable results were obtained in repeated experiments

**Fig. 3:**
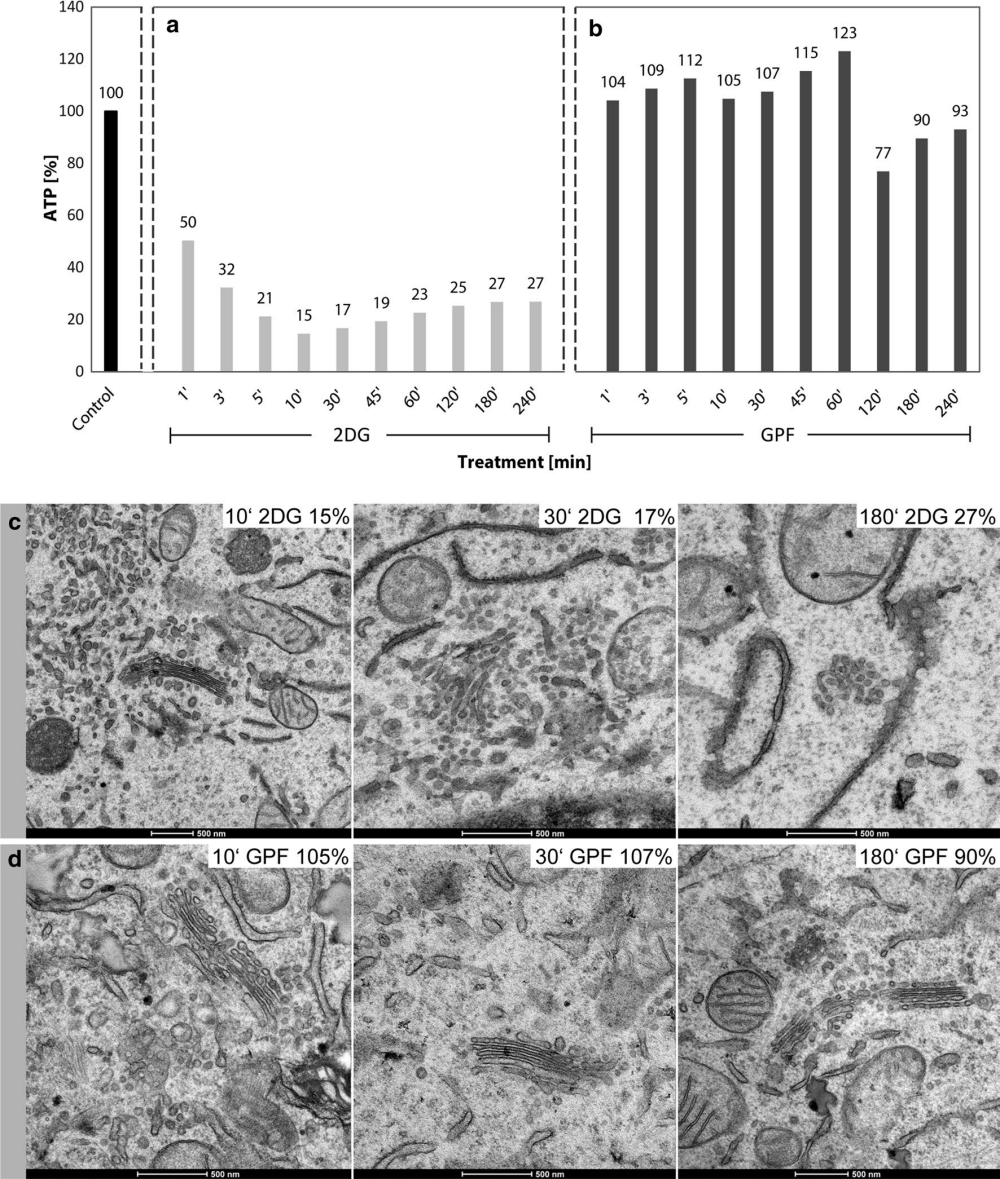
**a**, **b** Percentage ATP-values are shown as obtained after different times of culture in either glucose-pyruvate-free medium (GPF) containing 50 mM 2DG or in GPF lacking 2DG, respectively. The curve of declining ATP-values in **a** indicates that the main ATP-reduction occurs within the first 10 min of treatment. By contrast to the considerably reduced ATP-levels measured in the 2DG-treated cell cultures, the results obtained with the cultures grown in GPF without 2DG show ATP-values comparable to the controls or slightly reduced at the later times of treatment. The data are taken from one representative experiment; repeated experiments yielded comparable results. **c**, **d** The Golgi apparatus morphologies of cells cultured for 10, 30 and 180 min in GPF with or without 2DG, respectively; the respective ATP-values are additionally indicated in the right upper corner of the pictures. **c** Alterations of the Golgi apparatus stack are hardly visible after 10 min of 2DG-treatment; the stack appears disorganized and in part changed in a loosely arranged Golgi body after 30 min; a Golgi body composed of densely packed membrane compartments is seen after 180 min of treatment. **d** By contrast, cells cultured in GPF without 2DG show unchanged Golgi apparatus stacks comparable to those of control cells. In all pictures, RER-cisternae are found nearby the Golgi stacks exhibiting denser contents in the 2DG-treated cells (**c**) than in those cultured without 2DG

Incubation of cultured cells in a glucose-pyruvate-free medium (GPF) containing 2DG effects a rapid reduction in the cells’ ATP-levels and prominent reorganizations of the Golgi apparatus (Figs. 1, 2, 3). The ATP-levels decline within the first minutes after 2DG-administration to approximately 15–20% of those of the controls or even lower values and stay low during continued treatment (Fig. 3a). Simultaneously, regular Golgi apparatus stacks with typical flattened cisternae in parallel organization disappear and are replaced by bodies composed of loosely arranged vesiculo-tubulo-cisternal membrane compartments, as depicted electron microscopically by studies of thin sections (Fig. 1a, b and inserts) and three-dimensionally by electron tomography (Fig. 2a–c). For this work, we used cells of the well-established human HepG2 hepatoblastoma cell line (Aden et al. 1979; Knowles et al. 1980; Schwartz et al. 1981; Zannis et al. 1981; López-Terrada et al. 2009). Since tumor cells differ from nontumor cells in that they show generally higher rates of glycolysis (Cori and Cori 1925; Warburg 1930, 1956a, b; reviewed by Ferreira 2010), they are most suitable for studying 2DG as anti-metabolite of glucose (for review, e.g., Kang and Hwang 2006). We studied high-pressure frozen cells as well as chemically fixed cells and did not find differences in the changes of the Golgi apparatus induced by 2DG. In preliminary examinations, we tested the basic culture conditions, studied possible influences of different 2DG-concentrations and compared the 2DG-results with those obtained by starvation of the cells.

### Basic analyses

#### The 2DG-effects on ATP-levels and Golgi apparatus morphologies are independent of the concentrations used

Since, in the literature, the concentrations of 2DG used vary strongly depending on the cell type and scientific interest (Table 1), we tested the effects of different 2DG-concentrations on the cells of our HepG2 cultures. ATP-levels and the cells’ morphologies were analyzed after 45 min and 5 h of treatment with 10, 25, and 50 mM 2DG. No differences between the three concentrations were found neither concerning the 2DG-induced morphological Golgi apparatus changes nor the ATP-reductions (Fig. 2d).

**Table 1:** Overview of cell lines and 2DG-concentrations used in various scientific studies

Cell line	Source	Concentration of 2DG (mM)	References
1420	Pancreatic cancer (*Homo sapiens*)	4	Xi et al. (2011)
A549	Lung carcinoma (*Homo sapiens*)	5 and 100	Djuzenova et al. (2009)
		100	Wu et al. (2007)
BAEC	Aortic endothelial cells (Bovine)	5	Wang et al. (2011)
BT-549	Breast cancer (*Homo sapiens*)	4-12	Aft et al. (2002)
C2C12	Myoblast (*Mus musculus*)	25	Hong and Hagen (2015)
DU145	Prostate cancer (*Homo sapiens*)	20	Li et al. (2015)
GaMG	Glioblastoma (*Homo sapiens*)	5 and 100	Djuzenova et al. (2009)
GIST-T1 GIST48 GIST48B GIST430 GIST882	Metastatic gastrointestinal stromal tumor (*Homo sapiens*)	0.01–10	Mühlenberg et al. (2015)
GL15	Glioblastoma (*Homo sapiens*)	5	Zhang et al. (2006)
H460	Large cell lung cancer (*Homo sapiens*)	100	Wu et al. (2007)
H1299	Nonsmall cell lung carcinoma, derived from metastatic site: lymph node (*Homo sapiens*)	1–100	Kobayashi et al. (2015)
HCT116	Colorectal cancer (*Homo sapiens*)	0.5–20	Ahadova et al. (2015)
		1–100	Kobayashi et al. (2015)
HEK293T	Embryonic kidney (*Homo sapiens*)	25	Hong and Hagen (2015)
HeLa	Cervix cancer (*Homo sapiens*)	1–10	Kobayashi et al. (2015)
		4–10	Lin et al. (2003)
		45	Maehama et al. (1998)
Hep-2 (CCL-23)	Epithelial cells from HeLa contaminant tissue (*Homo sapiens*)	10	Kavaliauskiene et al. (2015)
HepG2	Epithelial hepatoblastoma (*Homo sapiens*)	5	Zhang et al. (2006)
		25	Hong and Hagen (2015)
		50	Ranftler et al. (2015)
HMEC	Mammary epithelial cells (*Homo sapiens*)	20	Li et al. (2015)
HT29-D4	Colon carcinoma (*Homo sapiens*)	5	Zhang et al. (2006)
HT29 (HTB-38)	Colon adenocarcinoma (*Homo sapiens*)	10	Kavaliauskiene et al. (2015)
HT1080	Fibrosarcoma (*Homo sapiens*)	5 and 100	Djuzenova et al. (2009)
IGROV1	Ovarian carcinoma (*Homo sapiens*)	5	Zhang et al. (2006)
LN-229	Glioblastoma (*Homo sapiens*)	1.6–50	Wu et al. (2009)
MCF-7	Breast cancer (*Homo sapiens*)	0.5	Oladghaffari et al. (2015)
		4–12	Aft et al. (2002)
MDA-MB-231	Breast cancer (*Homo sapiens*)	20	Li et al. (2015)
MDA-MB-435	Melanoma (*Homo sapiens*)	10	Xi et al. (2011)
MDA-MB-468	Breast cancer (*Homo sapiens*)	4–12	Aft et al. (2002)
MDCK	Madin-Darby canine kidney epithelial (Dog)	25	Hong and Hagen (2015)
MSTO-211H	Lung mesothelioma (*Homo sapiens*)	5	Zhang et al. (2006)
NCI-H28	Lung mesothelioma (*Homo sapiens*)	5	Zhang et al. (2006)
NRK	Normal kidney (Rat)	50	del Valle et al. (1999)
PC3	Prostate cancer (*Homo sapiens*)	0.005	Jangamreddy et al. (2015)
		0–20	Jeon et al. (2015)
		20	Li et al. (2015)
RPMI-2650	Squamous cell carcinoma of the nasal septum (*Homo sapiens*)	4	Keenan et al. (2004)
SCC61	Head and neck squamous cell carcinoma (*Homo sapiens*)	5	Zhang et al. (2006)
SH-EP	Neuroblastoma (*Homo sapiens*)	15	Hagenbuchner et al. (2015)
SKBR3	Breast cancer (*Homo sapiens*)	0.5	Oladghaffari et al. (2015)
		4–12	Aft et al. (2002)
		10	Xi et al. (2011)
SKOV3	Ovarian carcinoma (*Homo sapiens*)	5	Zhang et al. (2006)
SQ2OB	Head and neck squamous cell carcinoma (*Homo sapiens*)	5	Zhang et al. (2006)
SVEC4-10	Lymphoid endothelial (Mus musculus)	0–20	Huang et al. (2015)
STA-NB1 STA-NB4 STA-NB8 STA-NB15	Neuroblastoma (*Homo sapiens*)	15	Hagenbuchner et al. (2015)
SW480 (CCL-228)	colorectal adenocarcinoma (*HOMO sapiens*)	10	Kavaliauskiene et al. (2015)
SW620	Colorectal cancer (*Homo sapiens*)	1–20	Muley et al. (2015)
T47D	Breast cancer (*Homo sapiens*)	20	Mustafa et al. (2015)
T98G	Glioblastoma (*Homo sapiens*)	1.6–50	Wu et al. (2009)
U87-MG	Glioblastoma-astrocytoma (*Homo sapiens*)	5 and 100	Djuzenova et al. (2009)
U251	Glioblastoma (*Homo sapiens*)	5	Zhang et al. (2006)

#### The effects of 2DG treatments are specific and differ to those caused by starvation

In order to answer the question, whether the effects of 2DG-treatment on the cellular ATP-levels and Golgi apparatus morphologies can also be achieved by starvation, we compared the results obtained with cells incubated in GPF containing 50 mM 2DG with those obtained after incubation of the cells in GPF without 2DG. The ATP-levels after incubation in GPF without 2DG for various time periods ranging from 1 to 240 min were comparable to those of controls or slightly reduced but were never found significantly depressed as seen after 2DG-treatment (Fig. 3a, b). The Golgi apparatus shows unchanged morphologies in the cells cultured in GPF (Fig. 3d), thus contrasting to the altered Golgi apparatus stacks, which are on display in Fig. 3c.

#### The ATP-lowering effects of 2DG can be inhibited by d-glucose

**Fig. 4:**
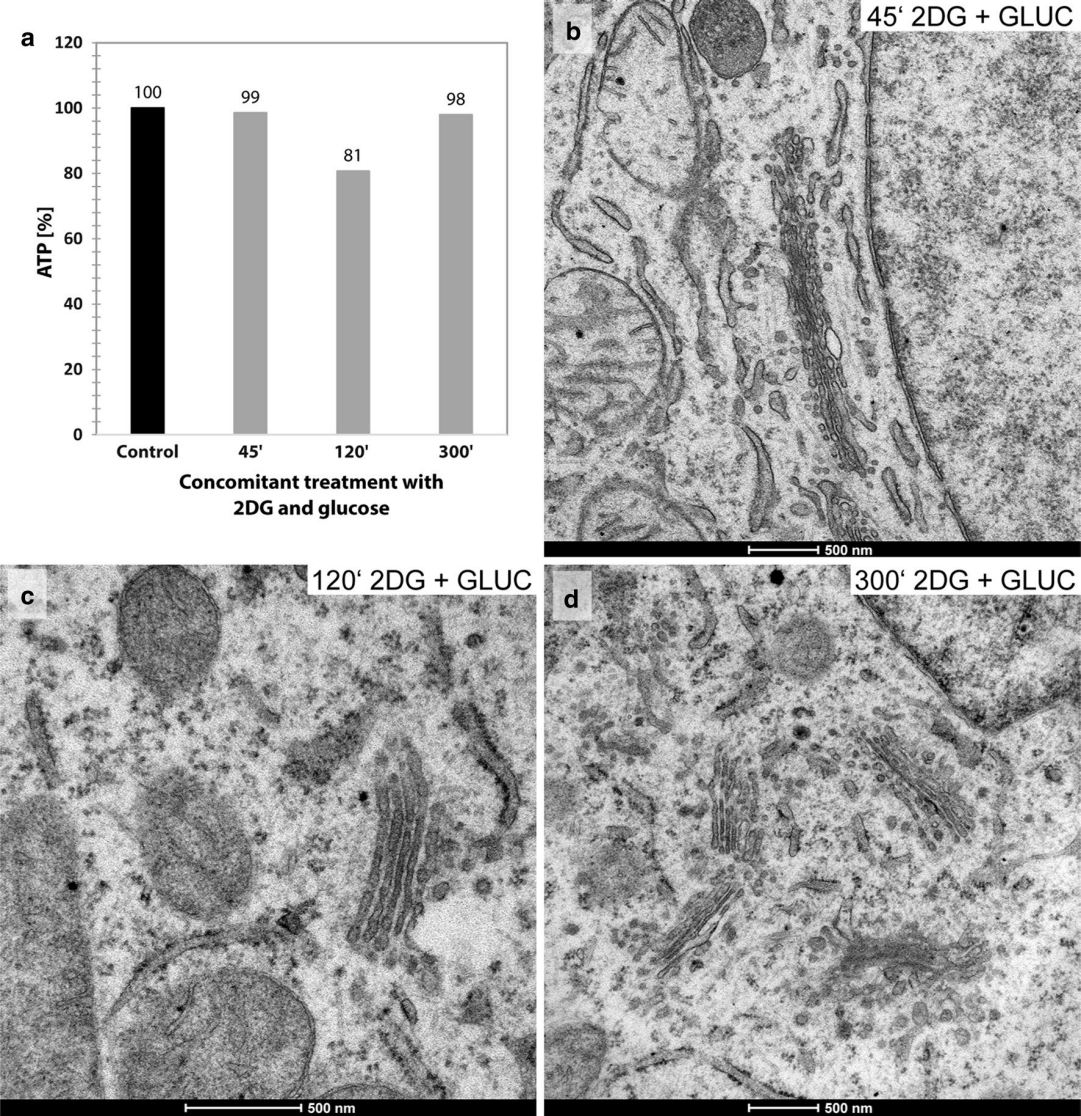
**a** Unchanged cellular percentage ATP-levels after 45, 120 and 300 min of concomitant treatment of the HepG2 cell cultures with 2DG (50 mM) and d-glucose (50 mM) indicating that the ATP-lowering effect of 2DG is inhibited. The likewise unchanged morphologies of the Golgi apparatus stacks, as they are found after 45, 120 and 300 min of concomitant treatment with 2DG and d-glucose, are shown in **b**, **c** and **d**

Concomitant treatments of the HepG2 cells with 2DG (50 mM) and d-glucose (50 mM) block the ATP-lowering effect of 2DG. This inhibition is effective at all times of treatment (Fig. 4a); the Golgi apparatus morphologies remain unchanged (Fig. 4b–d).

### Structural Golgi stack reorganizations

The main parts of the work focus on the structural changes in the Golgi apparatus taking place in response to 2DG-treatment and after its removal. The results underline that 2DG is a substance that allows a controlled disorganization and re-formation of this complex organelle thus providing insight into the dynamics of its architecture.

#### Initial 2DG-induced Golgi reorganizations take place within the stacks of cisternae

**Fig. 5:**
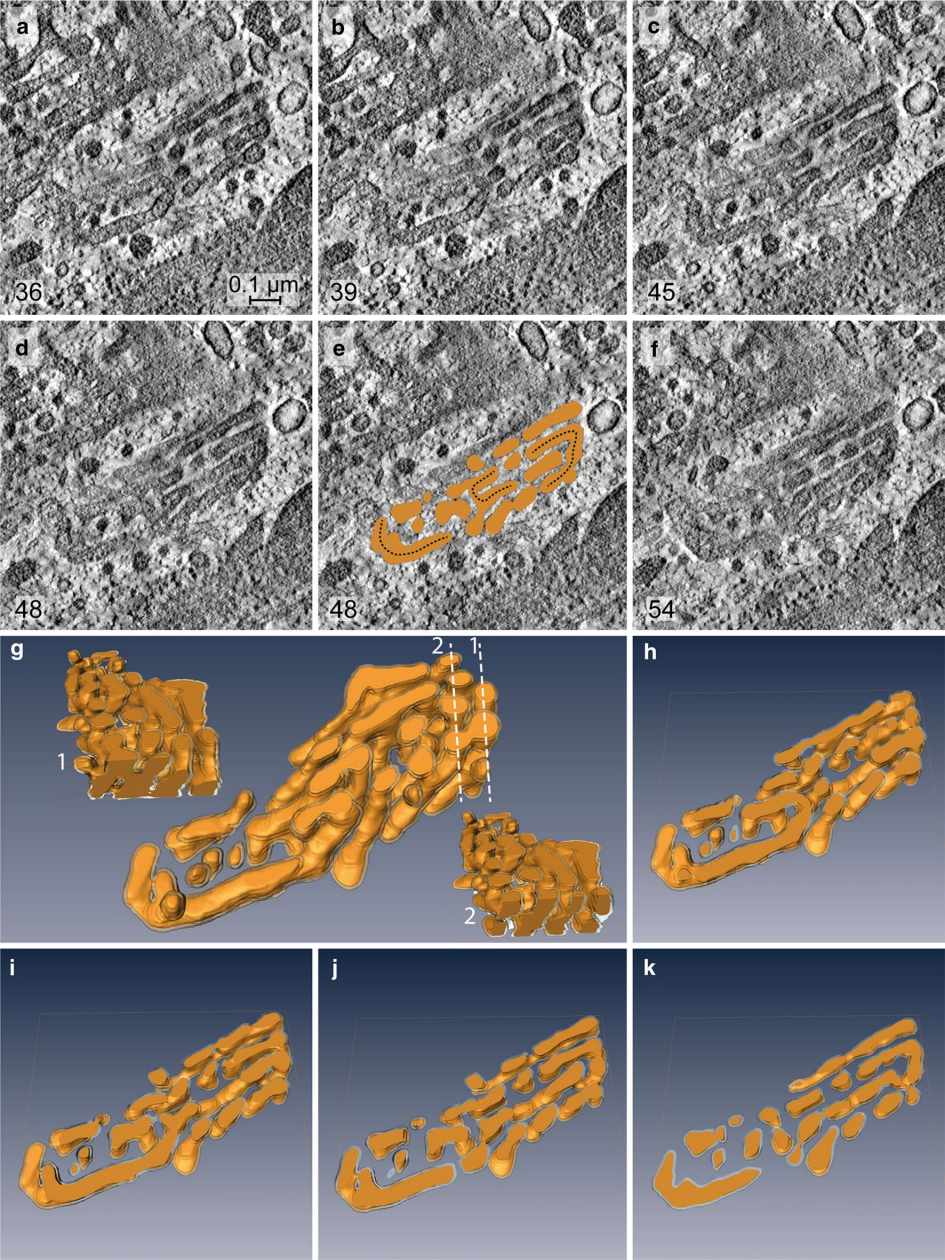
**a**–**f** Tomographic slices obtained from different levels of a Golgi stack reconstruction after 10 min of 2DG-treatment; the respective 3D-model is on display in **g**–**k**. Both the slices and the model, in part sectioned vertically (**g1** and **g2**) and horizontally (**h**–**k**), provide views inside the stack. The image shows that a parallel organization of cisternae still exists but is interrupted by arches, branches and wide pores resulting in the occurrence of reticular regions within the stack. Regularly ordered cisternae and reticular areas are located side by side, as is particularly clearly shown in **g**. In **e**, slice 48 is shown together with the respective horizontal section through the model, and the connecting arches present within the stack are accentuated by *dotted lines*. The *numbers* in the *left lower corner* of **a**–**f** indicate the respective slice numbers within the reconstructed stack. Volume of the calculated tomogram (*x*, *y*, *z*): 989 × 976 × 61 pixels, pixel size 1.84 nm

**Fig. 6:**
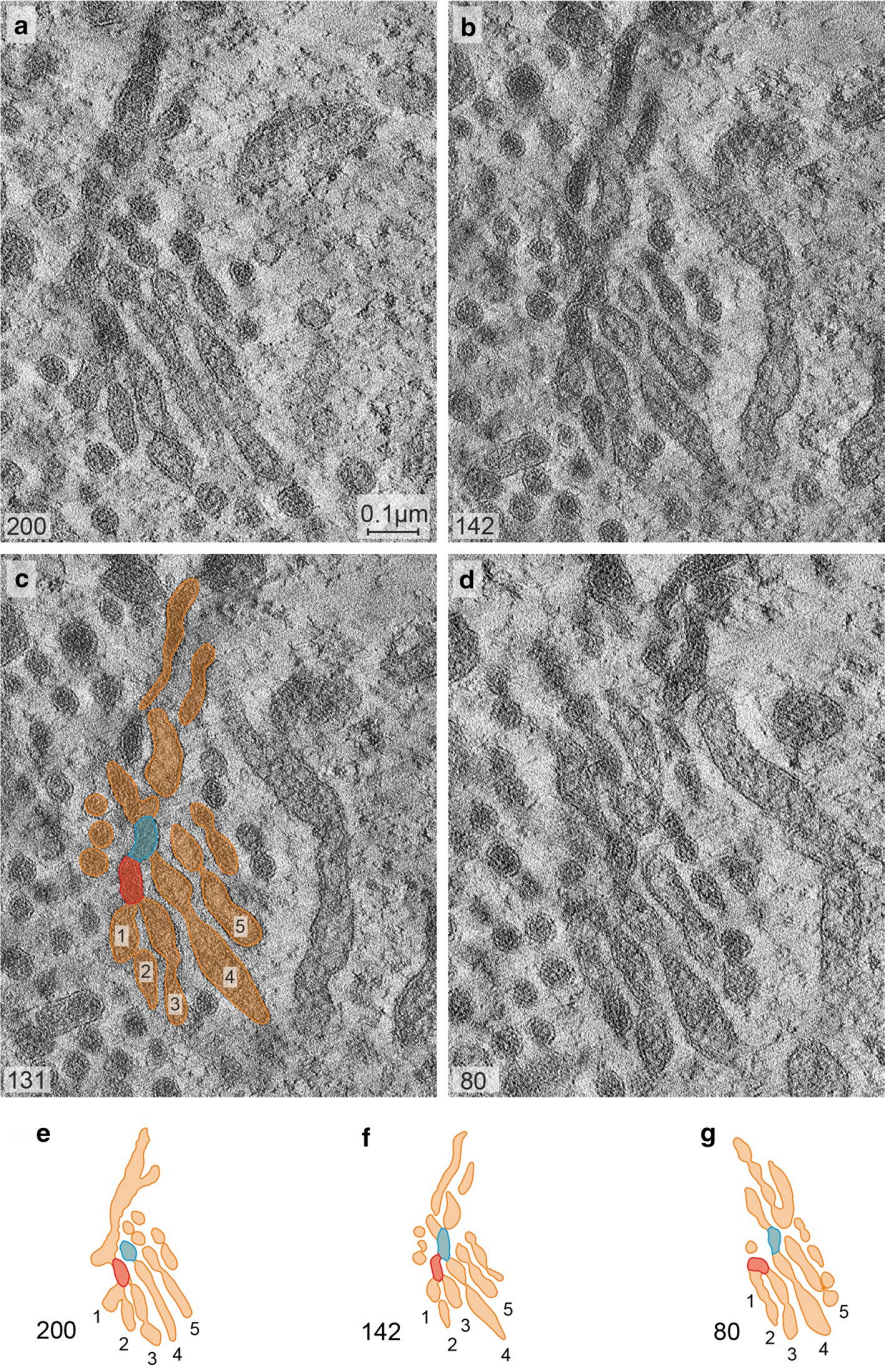
Tomographic slices in **a**–**d** obtained from different levels of a Golgi apparatus reconstruction at 15 min of 2DG-treatment show that different sites within a stack are interlinked by crossroads-like junctions, accompanied by frequent bifurcations. A representative area is on display in **a**, **b** and **d** and is further accentuated by means of drawings and by numbering some of the cisternae (**c**, **e**–**g**). Two different sites within the stack are labeled in *red* and *blue* and their spatial relationship traced within the reconstructed volume. The *colored* regions are found apart from each other in slice 200 (**a**, **e**); in slices 131 and 142, they can be seen joined forming parts of a crossroads-like junction (**b**, **c**, **f**), and they are separated again in slice 80 (**d**, **g**). The *numbers in the left lower corner* of **a**-**d** indicate the respective numbers of the slices. Volume of the calculated tomogram (*x*, *y*, *z*): 3.585 × 3.604 × 242 pixels, pixel size 0.39 nm

ATP-levels drop down to approximately 15–20% within the first 10 min and remain constantly low during all times tested (Fig. 3a). The early changes in the Golgi apparatus architecture in response to 10–15 min of 2DG-treatment can hardly be detected in thin sections (Fig. 3c, panel at the left-hand side) but they become clearly visible in the electron tomographic reconstructions (Figs. 5, 6). The virtual tomographic slices depicted in Fig. 5a–f and the different aspects of the respective model in Fig. 5g–k show that the regular parallel order of cisternae is interrupted by arches, branches and wide pores, and reticular regions emerge within the stacks that are situated side by side with regularly organized cisternae (Fig. 5g). Slices and drawings on display in Fig. 6 (panels a–d and e–g, respectively) show the presence of crossroads-like junctions that build up interlinked regions within the stack. In both cases, the 3D-tomographic analyses provide evidence that almost all parts of the stack are connected with each other. This is accentuated by a color code and by numbering some of the cisternae (Fig. 6).

#### With ongoing 2DG-treatment Golgi stacks disappear and are replaced by Golgi bodies

**Fig. 7:**
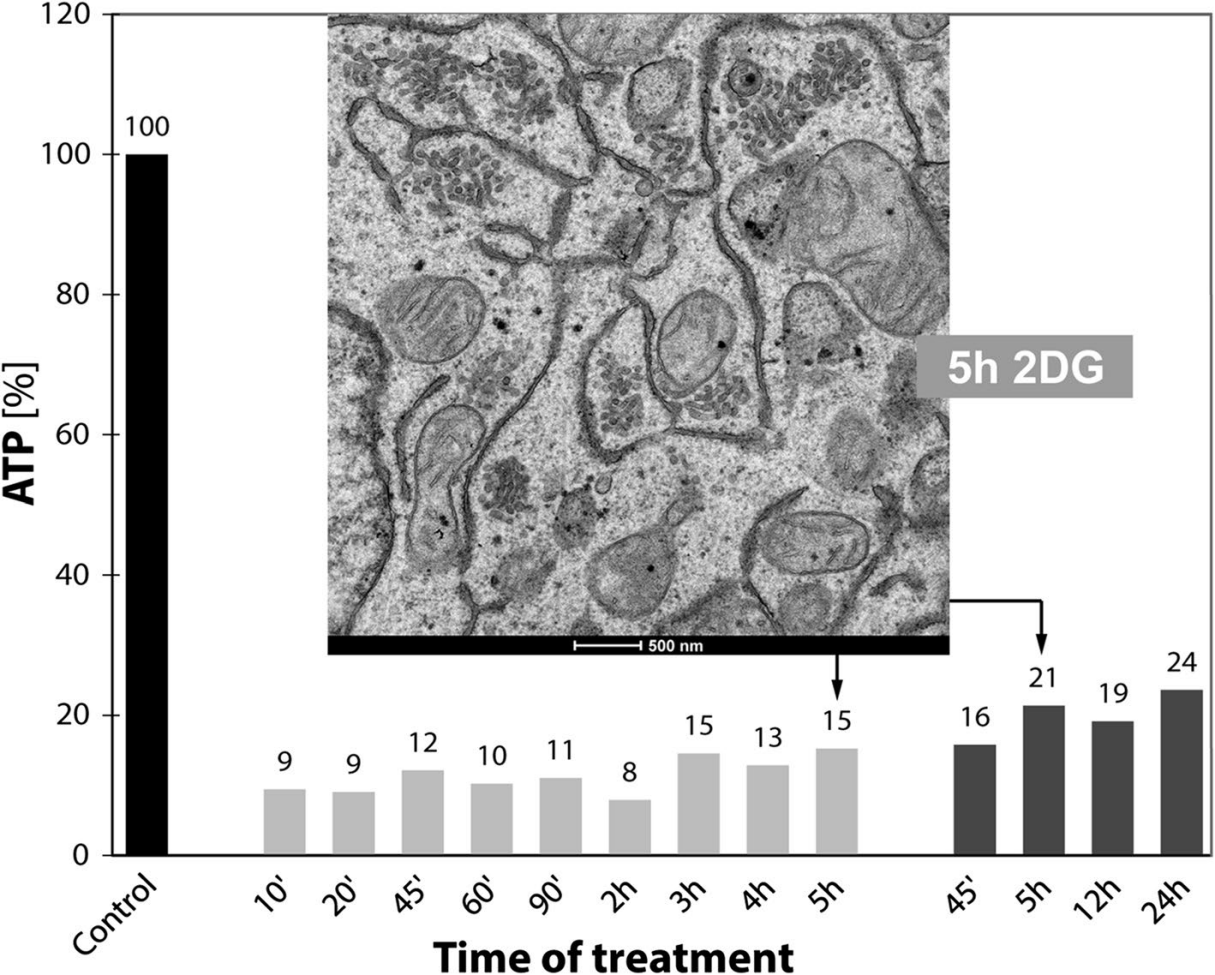
Percentage ATP-values after different short- and long-time 2DG-treatments are shown. The data are taken from two representative experiments and show that the ATP-concentrations are persistently depressed throughout the entire times of the experiments. The electron micrograph shows parts of the cytoplasm of a HepG2 cell treated with 2DG for 5 h. Regular Golgi apparatus stacks are lacking; instead, Golgi bodies with densely packed membrane compartments dominate closely adjacent to cisternae of the RER filled with electron dense contents

**Fig. 8:**
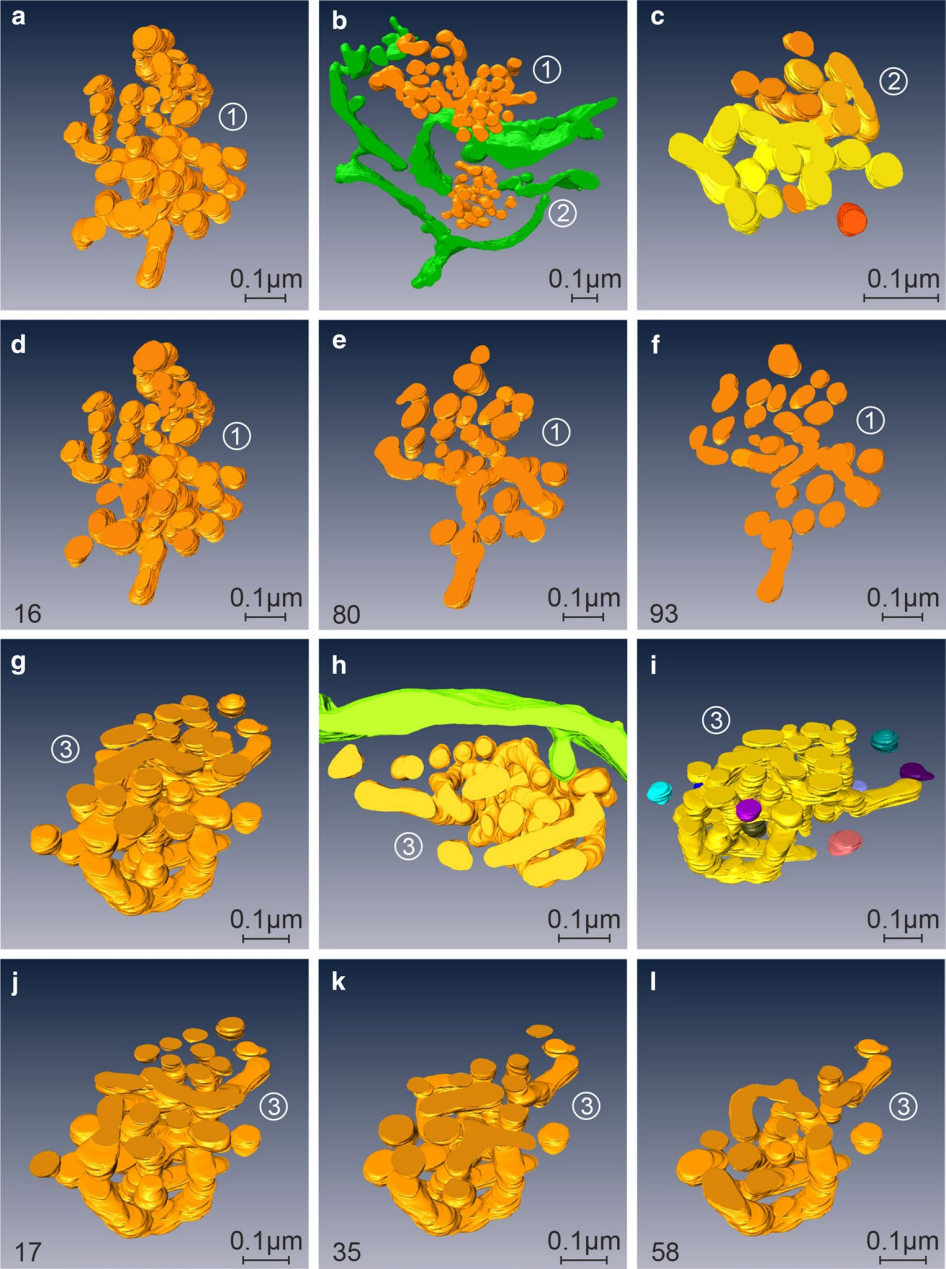
Panels depict various views of 3D-models of compact Golgi bodies (numbered 1, 2 and 3) after 60 min of 2DG-treatment. **b**, **h** Indicate the close relationship to the ER (colored in *green*); an ER-bud protruding into the body is shown in panel h. All 3 bodies are similarly composed of convoluted tubules, the loops of which can be identified in the models and are particularly well visible in the sections through the models, exhibited in **d**–**f** and **j**–**l**. The differentiated *coloring* of bodies 2 and 3 in **c** and **i** points to the connected parts of the bodies, the largest being highlighted in *yellow*. As seen in **i**, body 3 is almost completely composed of one continuous compartment. The *numbers in the left lower corner* of **d**–**f** and **j**–**l** indicate the respective positions of the sections through the model. Volumes of the calculated tomograms (*x*, *y*, *z*): **a**–**f** 4.004 × 3.892 × 310 pixels, pixel size 0,39 nm; **g**–**l** 3.977 × 3.887 × 370 pixels; pixel size 0.39 nm

With progressing 2DG-treatment, ATP-levels remain low (Fig. 3a); both short- and long-time treatments lead to similarly depressed cellular ATP-concentrations (Fig. 7). Concomitantly, regular Golgi stacks become reduced in number, disappear and get replaced by Golgi bodies, visible in thin sections in the electron microscope as loosely organized heterogeneous membrane compartments (Fig. 1b). Regular Golgi stacks are almost entirely lacking after 1-h treatment and Golgi bodies dominate. With continuing 2DG-applications, the character of the bodies alters. They become smaller and increasingly compact and glomerular (Figs. 3c, 7). Figure 3c shows the change in the organization: loosely arranged bodies at 30 min and a compact body at 180 min 2DG-treatment. The tomographic slices and models (Figs. 2c, 8a–l) providing 3D-views and insights into the interior of the bodies confirm the reorganizations. The early loosely arranged tubulo-cisternal Golgi body, accompanied by small vesicles (Fig. 2c), contrasts with the late compact bodies (Fig. 8), which represent distinct glomerular organelles with densely packed membranes and only few vesicles nearby. Sections through the 3D-models show that convoluted tubules build up these compact organelles (Fig. 8d–f, j–l). Large parts of the bodies are connected among each other. A remarkably close spatial relationship of the ER is shown in Fig. 8b and h, where an ER-bud protruding into the body is visible (Fig. 8h).

#### After 2DG-removal, Golgi stack re-formation takes place in a close spatial relationship to Golgi bodies

**Fig. 9:**
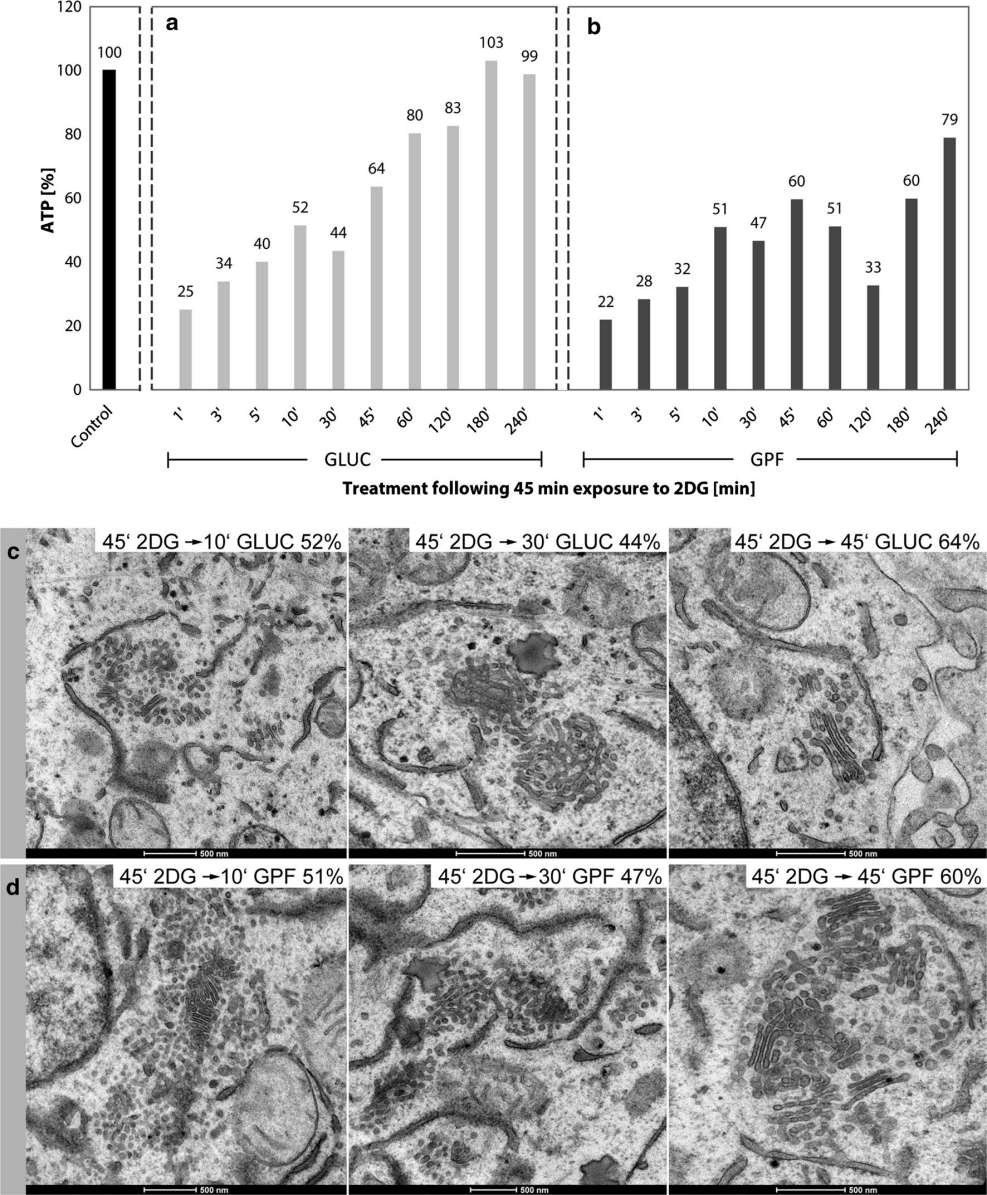
**a** Rising cellular ATP-levels at different times ranging from 1 to 240 min after removal of 2DG and incubation in a 50 mM glucose- and 1% pyruvate-containing DMEM. **b** The respective ATP-values obtained after 2DG-removal and incubation of the cells in glucose-pyruvate-free medium (GPF). A comparison of the diagrams in panels a and b makes it clear that ATP-replenishment also takes place without addition of glucose but is delayed. The pictures in **c**, **d** show the Golgi apparatus morphologies 10, 30 and 45 min after 2DG-removal and incubation in either glucose-containing or glucose-free medium, respectively. With either protocol, an increased regularity of the membranes and short cisternae in parallel orientation can be seen within the Golgi bodies as early as after 10 min. Mini-stacks are formed that are particularly well visible in the rightmost picture of **d**

**Fig. 10:**
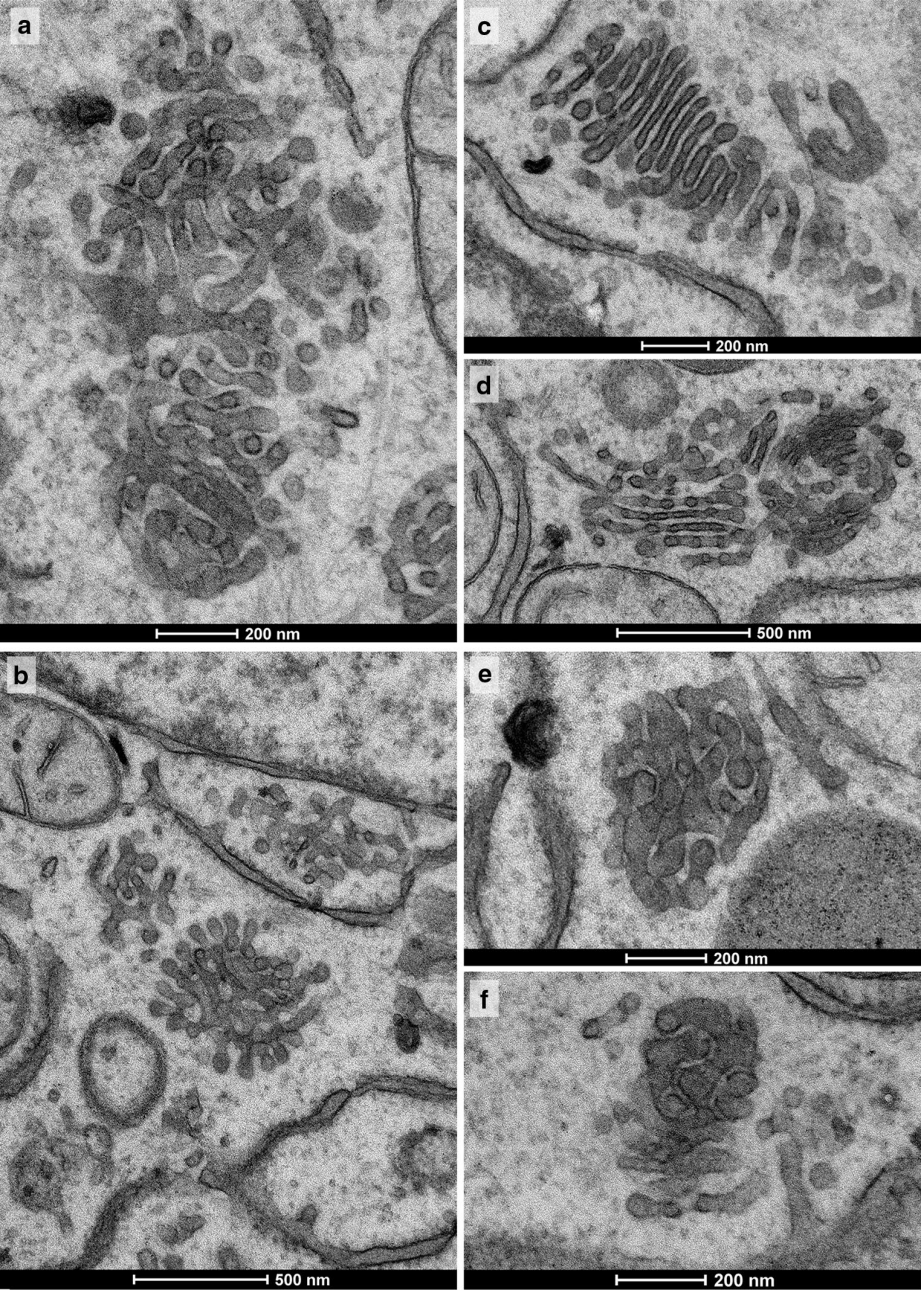
All panels show Golgi apparatus bodies after 2DG-removal following a 45 min treatment time and a 30 min subsequent incubation in GPF. Various types of Golgi bodies are on display, as they also may reside in cells side by side. The bodies shown in **a**, **b** are of tubular-reticular character; **e**, **f** particularly compact glomerular Golgi bodies with densely packed membranes, and **c** and **d** initial stack formations. **c** Multi-cisternal mini-stack, as is characteristic for this recovery period. The cisternae are conspicuously short, lack pores and are in a ladder-like arrangement. A combined body is shown in **d** consisting of a compact part with densely packed membranes on the *right-hand side*; in the stack on the *left-hand side*, pores can be seen and pairs of cisternae appear connected at their rims. Narrow, particularly regular inter-membrane spaces are apparent in the compact body shown in **f**

**Fig. 11:**
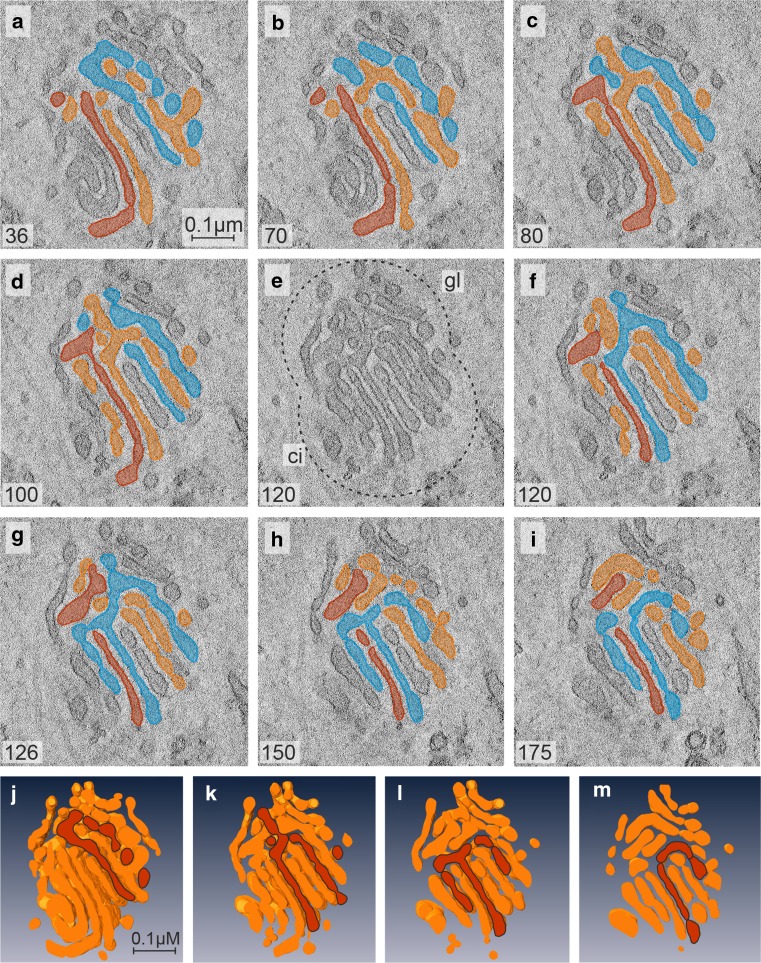
**a**–**i** and **j**–**m** Tomographic slices and pictures of the respective 3D-model of a re-forming Golgi stack located within a compact Golgi body. The investigated cells are obtained from a culture after removal of 2DG following 45 min of 2DG treatment and subsequent incubation in a glucose-pyruvate-free medium for 45 min. The body consists of a tubulo-glomerular and a cisternal part (in **e** highlighted by a *dotted line* and termed *gl* and *ci*, respectively). Almost all of the short cisternae of the mini-stack emerge from the tubulo-glomerular part of the body and are connected among each other via wide hanger-like arches in part extending from one to the other side of the stack. In the series of slices (**a**–**d**, **f**–**i**) and sections through the model (**j**–**m**) some of the cisternae are accentuated by *colors* to be able to follow up their extensions throughout the body more easily. Volume of the calculated tomogram (*x*, *y*, *z*): 3.223 × 2.847 × 200 pixels, pixel size 0.39 nm

Both the 2DG-induced ATP-decrease and the Golgi apparatus reorganizations are reversible. The cellular ATP-contents are rapidly replenished after removal of 2DG and incubation of the cells in medium containing 50 mM glucose and 1% pyruvate (Fig. 9a). The ATP-levels arise fast and reach those of controls after 60–180 min; simultaneously, regular Golgi apparatus morphologies re-appear (Fig. 9c). ATP-replenishment and reconstitution of regular Golgi apparatus stacks after removal of 2DG also take place in GPF, but are delayed under such conditions (Figs. 9b, d, 10, 11). In cells incubated in medium with 50 mM glucose, the ATP-levels increase rapidly (Fig. 9a) and regular Golgi apparatus stacks are found as early as after 45 min (Fig. 9c, picture in rightmost position), whereas ATP-replenishment takes place more slowly (Fig. 9b) and most of the Golgi stacks are at this time still in a state of re-formation, when glucose-free medium is used (Fig. 9d, picture in rightmost position). Bodies of densely compacted membranes (Fig. 10e, f) and multi-cisternal mini-stacks of short pore-less cisternae in a ladder-like arrangement (Fig. 10c) precede the re-appearance of regular Golgi apparatus stacks. In the compacted bodies narrow, particularly regular inter-membrane spaces are dominant (Fig. 10f). Mini-stacks are formed that are closely related to compact Golgi bodies as shown in the electron tomographic slices and sections through the model on display in Fig. 11. Almost all of the short cisternae of the forming mini-stack are connected among each other via complex continuities located in the tubulo-glomerular part of the body (Fig. 11a–i, j–m).

#### Golgi apparatus dis- and reorganizations correlate with the cellular ATP-concentrations

**Fig. 12:**
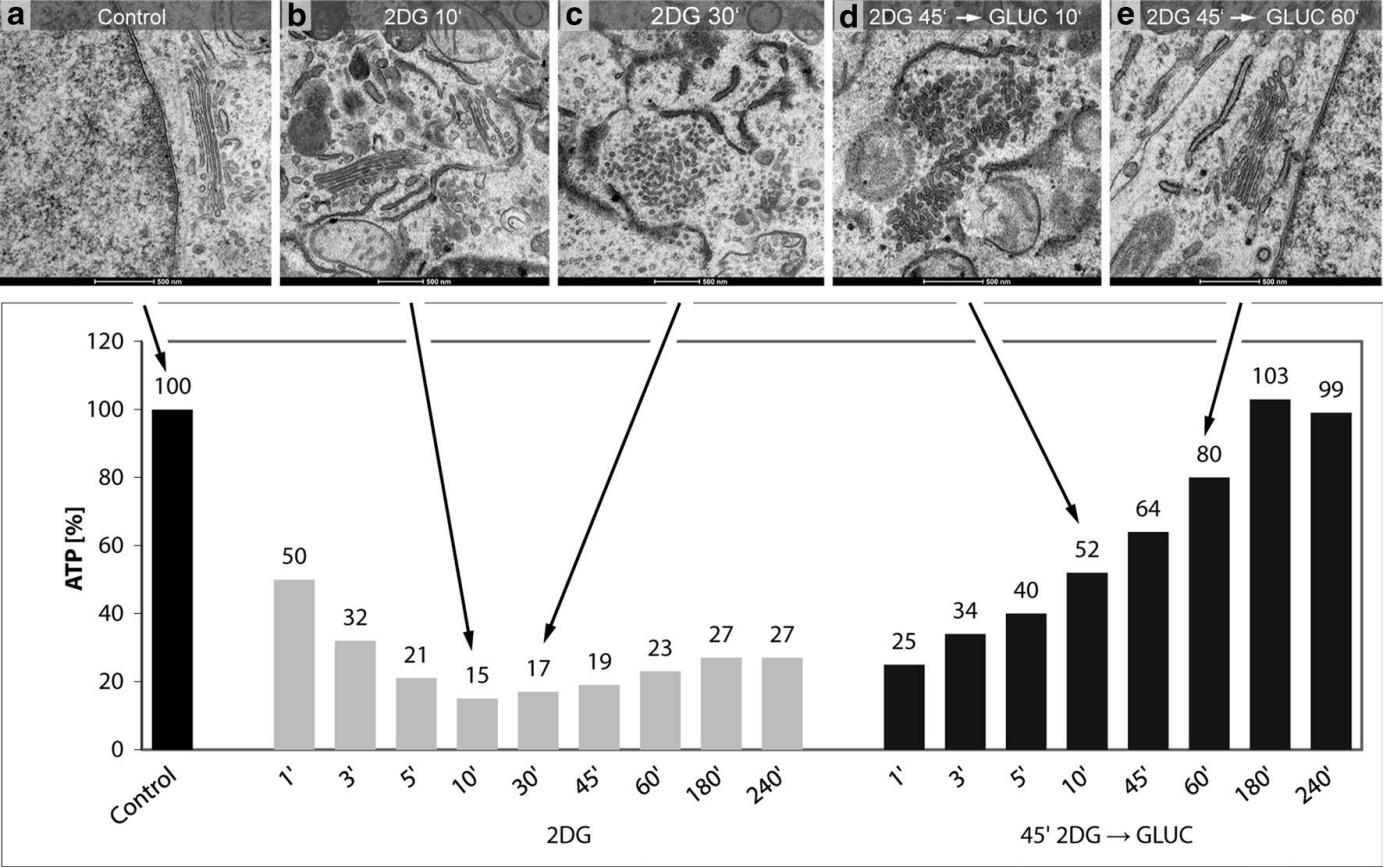
Artwork shows the results of correlative ATP-analyses and ultrastructural analyses highlighting the ATP-levels and the corresponding Golgi apparatus morphologies at 10 and 30 min of 2DG-treatment and 10 and 60 min after 2DG-removal and incubation in glucose-containing medium. **a** Regularly structured Golgi apparatus stack of a control cell. The results shown in **b** and **c** make evident that the processes leading to a replacement of the regular Golgi stacks by Golgi bodies occurs in a phase of low cellular ATP-concentrations. **d** The occurrence of mini-stacks within the Golgi bodies, indicating the beginning of Golgi apparatus re-formation, coincides with the ATP-level increase after removal of 2DG. The picture in panel **e** exhibits a control-like regularly structured Golgi stack after ATP-replenishment

The 2DG-induced reorganizations of the Golgi apparatus can be correlated with the changes in the cellular ATP-levels. We performed ATP-measurements at short intervals and electron microscopic analyses of cells of the same cultures in parallel. The results showed that the amounts of cellular ATP and the Golgi apparatus structures change concomitantly during both the ATP-decrease and the ATP-replenishment periods. A summary depicting the ATP-values of one representative experiment and characteristic electron micrographs obtained from cells at the same times of treatment is shown in the artwork of Fig. 12. In the experiment on display (see also Fig. 3a), the ATP-levels decline within 10 min to 15% of those of the controls (Fig. 12a, b). In parallel, the first changes occur within the Golgi apparatus stacks. The initial membrane reorganizations are inconspicuous and can be easily missed if only thin sections are analyzed, but they are evident in the three-dimensional reconstructions (cf. Figs. 5, 6). During the subsequent periods of 2DG-treatment from 10 min up to 4 h, the ATP-levels remain low ranging between 15 and 27% of those of the controls; these periods are characterized by the absence of regular Golgi apparatus stacks and the dominance of vesiculo-tubulo-glomerular Golgi bodies (Fig. 12c). The increase of the ATP-levels in the ATP-replenishment period after removal of 2DG and addition of glucose and pyruvate to the culture medium is accompanied by a re-formation of regular Golgi apparatus stacks. In the experiment shown (see also Fig. 9a), the ATP-levels reach 52% of the controls after 10 min of incubation in glucose-containing replenishment medium; at this time, the Golgi apparatus stacks are not yet completely re-formed, but mini-stacks are visible within the Golgi bodies (Fig. 12d). After 1 h, the cellular ATP has reached 80% of that of controls and regularly structured Golgi stacks dominate (Fig. 12e).

## Discussion

The nonmetabolizable glucose analogue 2DG is used in experimental cell biology as well as in medical and clinical domains. Here, we show that 2DG also is a greatly valuable agent for analyzing organization and dynamics of the Golgi apparatus. With the usage of 2DG, the Golgi apparatus can be disorganized and subsequently again reorganized, thus permitting a close view of the processes of its dissociation, the remodeling of the stacks of cisternae, formation of Golgi bodies that replace the stacks and eventually the stacks’ re-formation.

Our measurements taken at frequent intervals indicate that addition of 2DG to the glucose- and pyruvate-free culture medium causes a rapid ATP-decrease within 10–15 min to some 15–20% of the control levels or even lower, remaining low over the entire time of treatment, whereas the ATP-contents rise again, as soon as 2DG is removed. The cells adapt to the altered environment and respond with structural changes that all are reversible after removal of the substance. Such investigations using 2DG are highly interesting not only concerning analyses of both Golgi stack dissociation and re-formation, but as they provide insights into the cells’ response to metabolic stress at the same time.

### Golgi stack dissociation and formation of Golgi bodies

In multiple publications, function-dependent Golgi apparatus changes, alterations during mitosis and in diseased cells and effects of Golgi-disturbing substances have been reported and described as Golgi apparatus dissociation, disassembly, breakdown, vesiculation or fragmentation (e.g., Rabouille and Warren 1997; Dinter and Berger 1998; del Valle et al. 1999; Wang and Seemann 2011; Villeneuve et al. 2013; Dong et al. 2014; Haase and Rabouille 2015; Machamer 2015; Schuberth et al. 2015; Kaneko et al. 2016); in most cases, the descriptions reflect the fluorescence microscopic patterns or are obtained by electron microscopy of thin sections. In our work, for the first time, 3D-electron tomography was used for studying the effects of a Golgi-disturbing substance. The results show that the Golgi apparatus does not simply “break down” under the influence of 2DG but that its compartments are remodeled leading to a disappearance of the organization of the stacks and their replacement by Golgi bodies. The reorganizations are not sudden events but take place within 30–60 min and more. Initial signs, however, recognized by 3D-analyses only can be seen within the first 10–15 min of treatment coinciding with the fast reduction in the cellular ATP-concentrations. Electron tomography made visible that early membranous intra-stack networks consist of arches, branches and crossroads-like structures connecting sites within the stacks located at a distance from each other. Their structures and extensions suggest that they could have a role in signaling (Cancino et al. 2014; Luini and Parashuraman 2016) or open novel traffic routes, which might be important during such early times of Golgi apparatus remodeling, when the stacks of cisternae increasingly disappear and Golgi bodies become apparent.

How membranes and contents in the secretory and endocytic systems traverse the Golgi apparatus stacks en route to their final destinations, thereby becoming modified, is one of the most debated questions in the areas of cellular transport and traffic. Different transport mechanisms, including vesicular traffic, membrane maturation and transport via membrane continuities and contact sites, have been shown and apply to different types of cells, depending on the molecules and materials to be transported but are possibly active in the same cell side by side (e.g., Trucco et al. 2004; Mironov et al. 2013; Glick and Nakano 2009; Pfeffer 2013; Pellett et al. 2013; Rizzo et al. 2013; Lavieu et al. 2013, 2014; Rothman 2014; Beznoussenko et al. 2014, 2016; Lee et al. 2014; Cheung et al. 2015; Nakano 2015; Dancourt et al. 2016). The reorganizations in response to 2DG show an increasing appearance of membrane continuities and connections, which start in the early phases of treatment with the occurrence of intra-stack networks and proceed with the formation and further remodeling of the Golgi bodies. Intercisternal connections, although they are not common, can be seen in unperturbed cells as well, mainly in connection with the formation of pathways for transport and traffic (e.g., Trucco et al. 2004; Mironov et al. 2013; Beznoussenko et al. 2014); our findings with 2DG at the very early times of treatment might express similar dynamics. However, the architectures are different; comparable structures with crossroads-like connections as seen in our 2DG-treated cells are not known to occur in unperturbed cells. It is remarkable, how many different cellular conditions lead to a disassembly or “breakdown” of the Golgi apparatus but little is known about the detailed courses of the stacks’ dissociations and whether there exists a general principle for the changes and those occurring during mitosis, in diseased cells and in response to treatments with various drugs are comparable with the 2DG-effects shown here. Interestingly, our 3D-results show continuing reorganizations of the Golgi bodies; the primarily formed bodies consisting of loosely arranged branched tubulo-cisternal and vesicular compartments are intermediates and replaced by compact organelles of densely packed convoluted tubules with only few vesicles nearby. The presence of vesicles accompanying the early Golgi bodies suggests that vesicular traffic into and out of the bodies takes place during these early times. Vesicles and other transport carriers might be derived from the ER and reflect the regular ER-Golgi pathway (for review Rothman 2014); frequently seen close proximities of Golgi bodies to sheets of the ER might reflect such a path, although this route is presumably impaired due to the 2DG effects on N-glycosylation, as discussed below. Vesicular structures might furthermore originate from the endocytic system (Vetterlein et al. 2002; Pavelka et al. 2008) or represent buds or fragments of the transforming Golgi compartments. The reduction in vesicles over the course of time can be considered, at least in part, to be connected with processes leading to their fusion and uptake into the Golgi bodies; such processes might contribute to the transformations of the early loosely arranged bodies, rich in vesicles, to the late compact organelles, in which vesicles are almost entirely lacking. On the other hand, vesicles may decrease in number through elimination by autophagy, which has been shown to be enhanced by 2DG-treatment (Wu et al. 2009; Wang et al. 2011; Xi et al. 2011; Jeon et al. 2015). Moreover, it should not be ignored that 2DG influences the early secretory pathways, because the impairment of N-glycosylation leads to an accumulation of miss-folded proteins in the ER. This becomes visible by electron microscopy, since the accumulated proteins appear as dense luminal ER-contents. The resulting impediment of the secretory transport and the impaired release from the ER might be another reason for the reduced number of vesicles nearby the compact Golgi bodies. The bodies’ different characters at early and late times, with abundant and rare vesicles, respectively, suggest that vesicular traffic into and out of a Golgi body declines with time. Our 3D-tomography analyses, on the other hand, show that the late compact Golgi bodies mainly consist of continuous convoluted tubules, which could provide alternative pathways for transport and substitute for traffic via vesicles.

### Golgi stack re-formation

In this work, for the first time, the reconstitution of a Golgi stack is analyzed by using 3D-electron microscopy; the results show a close spatial relation of the newly forming stacks of cisternae and compact tubulo-glomerular Golgi bodies. It is notable that the initially visible stacks are mini-forms containing very short cisternae that usually lack pores and are in a ladder-like arrangement; they often appear in a combined organelle consisting of a tubulo-glomerular part with densely packed membranes and a cisternal mini-stack part. The 3D-studies provide evidence that almost all of the short cisternae of the mini-stack emerge from the tubular-glomerular part of the body and almost all parts of the body are interconnected. This architecture prompts the consideration that the densely packed tubular membranes of the body may represent a membrane reservoir from which the membranes emerge for the formation, enlargement, and elongation of the cisternae of the mini-stack. The wide branched arches connecting the different sides and various sites of the body might constitute connecting roads for traffic across the re-forming stacks. They disappear with time, just as the tubulo-glomerular Golgi bodies disappear with the concomitant re-appearance of regular Golgi stacks. Obviously, neither structure is required any longer after the final formation of a regular Golgi stack and after the restart of other traffic mechanisms.

The particularly densely packed membranes and the narrow, regular inter-membrane spaces seen in some of the Golgi bodies (e.g., Figure 10f) point to possible membrane contact sites, where tethers, stacking proteins and/or signaling sites (Cancino et al. 2014; Rabouille and Linstedt 2016; Cheung and Pfeffer 2016; Zhang and Wang 2016; Levine and Patel 2016; Luini and Parashuraman 2016) might have important roles in the re-formation of the Golgi stacks and provide sites for lipid transfer.

### Correlation of Golgi morphologies and cellular ATP-levels

Our findings point to a close relation between the 2DG-induced structural alterations of the Golgi apparatus and the changes of the cellular ATP-concentrations, which are evident in all phases of the experiments. In the first phase of 2DG-treatment, when ATP drops, intra-stack-networks occur, and the stack organization loses its regularity; in the phases of continuous 2DG-treatment at consistently low ATP-levels, regular stacks are not apparent but are replaced by tubulo-glomerular Golgi bodies; in the ATP-replenishment-phase after 2DG-removal, the stacks re-form. These findings suggest that the highly organized classic Golgi stack architecture is impeded, while the cells are exposed to 2DG and the cellular ATP-concentrations are low, but the Golgi apparatus is preserved in the more simple form of Golgi bodies, in which continuous convoluted tubules might provide pathways for traffic. However, Golgi bodies can sustain functionalities, as recent studies in our laboratory have shown (Meisslitzer-Ruppitsch et al. 2011; Ranftler et al. 2015).

Although our results show that changes to the Golgi apparatus correlate with the cellular ATP-values, it cannot be concluded that the 2DG-effected Golgi disorganizations and formations of Golgi bodies are necessarily the results of the lowered cellular ATP-concentrations and impaired ATP-dependent cell functions. The Golgi apparatus and ATP-changes might occur in parallel, independently of each other, and there might be other actions of 2DG accounting for the Golgi stack reorganizations. In particular, it should be considered that a disrupted transport in the secretory systems due to the 2DG-interferance with N-glycosylation and an impaired release from the ER, leading to a reduction in the anterograde flow arriving at the *cis* Golgi side, might affect the Golgi stacks’ structures. Other 2DG-effects that might have an impact on the Golgi architecture comprise those on cellular lipids. It has recently been shown that 2DG alters the levels and species compositions of several lipids (Kavaliauskiene et al. 2015). This might affect membrane properties, possibly altering *trans*-membrane area asymmetries (Beznoussenko et al. 2015) and influence vesicle selection at the entrance of the Golgi apparatus (Magdeleine et al. 2016); both might contribute to structural changes and altered Golgi apparatus architectures.

### Summary

**Fig. 13:**
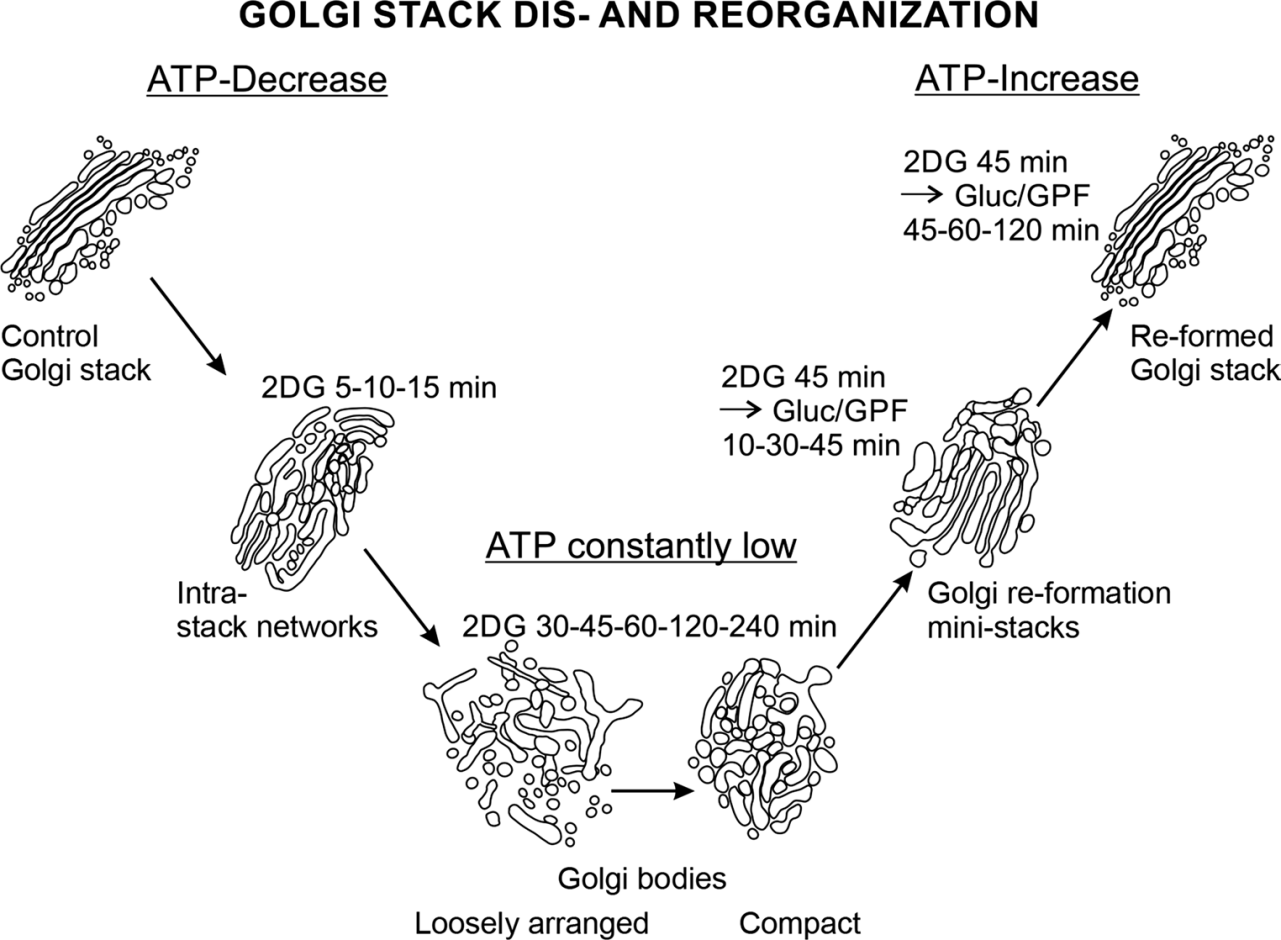
Summary of the dynamics of a Golgi apparatus stack during ATP-decrease in response to 2DG-application, upon constantly low ATP-levels during continued 2DG-treatment, and during ATP-replenishment after 2DG-removal

In conclusion, 2DG can be used for studying courses of Golgi stack remodeling. The changing architectures visualized in this work and summarized in Fig. 13 reflect Golgi stack dynamics that may be significant for basic cell physiologic and pathologic processes and help to learn, how cells respond to conditions of stress.
